# Effect of Copper-Coated Steel Fibers on the Electrical Conductivity of Concrete Under Multiple Influencing Factors

**DOI:** 10.3390/ma19214587

**Published:** 2026-10-29

**Authors:** Yixuan Zhao, Fuxue Sun, Zhongkun Wang, Yunhui Zhu

**Affiliations:** 1 College of Civil Engineering and Architecture, Wenzhou University, Wenzhou 325000, China; zyx13566196913@163.com (Y.Z.); sunfuxue@wzu.edu.cn (F.S.); wangzhongkun@wzu.edu.cn (Z.W.); 2 Key Laboratory of Engineering and Technology for Tideland Reclamation and Life-Cycle Intelligent Monitoring of Zhejiang Province, Wenzhou 325035, China; 3 Wenzhou Engineering Technical Research Center on Building Energy Conservation and Emission Reduction & Disaster Prevention and Mitigation, Wenzhou 325000, China; 4 College of Architecture and Energy Engineering, Wenzhou University of Technology, Wenzhou 325035, China

**Keywords:** copper-coated steel fiber, concrete, electrical resistivity, testing voltage, multifactor analysis

## Abstract

This study investigates the electrical response of concrete containing copper-coated steel fibers (CCSFs), with particular attention to the effects of voltage, which are insufficiently considered in conventional resistivity measurements. An optimized resistivity measurement method was developed to systematically evaluate the effects of CCSF volume fraction, water-to-cement ratio, temperature, and applied voltage on the electrical resistivity of CCSF concrete. The results showed that increasing the CCSF volume fraction from 0% to 3%, the temperature from −20 °C to 80 °C, and the applied voltage from 10 V to 60 V reduced the electrical resistivity by 87.18%, 92.99%, and 80.02%, respectively, whereas increasing the water-to-cement ratio from 0.35 to 0.55 resulted in a smaller reduction of 26.74%. The CCSF content and water-to-cement ratio primarily affected the baseline resistivity, while having limited effects on the normalized temperature response. The applied voltage also affected the absolute resistivity values, and the sensitivity of resistivity to applied voltage increased with increasing CCSF volume fraction. These findings indicate that applied voltage is a key parameter affecting both the electrical response of CCSF concrete and the comparability of resistivity measurements. This study provides experimental evidence for optimizing resistivity testing conditions and evaluating conductive concrete performance under complex environments.

## 1. Introduction

Concrete is one of the most widely used materials in civil engineering owing to its favorable mechanical properties and low cost [1]. With the growing demand for intelligent and multifunctional structures, the limitations of conventional concrete in applications such as stress monitoring [2,3,4], structural damage identification [5,6], electrical deicing [7], and electromagnetic shielding [8,9] have become increasingly apparent. These functions generally require a certain degree of electrical conductivity. However, ordinary concrete lacks continuous electron transport pathways, and its electrical conduction primarily relies on ion migration through the pore solution [10,11]. Consequently, its relatively high electrical resistivity limits its suitability for these functional applications. Improving electrical conductivity while maintaining the basic mechanical properties of concrete, together with understanding the factors governing its electrical behavior, is therefore essential to the development of electrically conductive cementitious composites.

The principal approach to improving the electrical conductivity of concrete is to incorporate conductive components into the matrix, thereby creating additional charge transport pathways and reducing electrical resistivity [12,13,14]. Commonly used conductive components can be classified as carbon-based materials [2,6,15], metal-based materials [9,13], and hybrid conductive materials [16,17]. However, some of these materials are difficult to disperse or may adversely affect the properties of the matrix [15,18]. Steel fibers, in contrast, combine mechanical reinforcement with electrical conductivity and offer favorable dispersibility and compatibility with practical construction processes, leading to their widespread use in engineering applications [19,20]. In particular, the copper coating on copper-coated steel fibers (CCSFs) may enhance both the electrical conductivity of individual fibers and conduction through contacts between fibers. CCSFs therefore have the potential to form conductive networks while improving mechanical properties [13].

Nevertheless, CCSF concrete is a multiphase composite whose electrical conductivity depends on multiple factors [11,21,22], with potential interactions among them. Existing studies have primarily focused on the effects of individual factors on electrical conductivity [9,13,21,22]. In practical engineering environments, however, concrete is typically subjected to several factors simultaneously. Studies of individual factors alone therefore cannot fully characterize the electrical response of CCSF concrete. Further investigation is needed to understand how its electrical conductivity varies under the combined influence of multiple factors.

In addition to material composition and environmental conditions, preliminary tests conducted in this study, together with an analysis of the literature [23], indicate that the electrical resistivity of CCSF concrete decreases as the applied voltage increases. However, this behavior has received limited attention in previous studies [2,22,23,24], and the influence of applied voltage on specimen resistivity has often been insufficiently considered in theoretical analyses and measurement procedures. Some studies have incorporated a resistor of known resistance into the measurement circuit [25,26] or adopted a four-electrode method [4,14,25,26]. In such configurations, differences in specimen resistance can lead to variations in the actual potential difference across the measurement region, thereby affecting the comparability of resistivity measurements. Existing measurement methods therefore need to be refined to control applied voltage as a key parameter, improve comparability across specimens, and clarify its influence on the electrical resistivity of CCSF concrete.

Accordingly, this study investigates CCSF concrete, with particular emphasis on its resistivity response to applied voltage. Specimen preparation and resistivity measurement methods are optimized to systematically examine the effects of CCSF volume fraction, water-to-cement ratio, temperature, and applied voltage on electrical conductivity. Variations in electrical resistivity are evaluated using sensitivity coefficients and normalization to characterize the electrical response of CCSF concrete under the influence of multiple factors.

## 2. Materials and Methods

### 2.1. Raw Materials and Mixture Proportions

Ordinary Portland cement (P·O 42.5), manufactured by Yueqing Conch Cement Co., Ltd. and purchased in Wenzhou, China, was used as the binder. Its properties met the relevant requirements of Chinese standard GB 175—2023. The aggregates comprised coarse aggregate with particle sizes ranging from 2.36 to 9.50 mm and fine aggregate consisting of sand conforming to the Zone II grading requirements. The coarse-to-fine aggregate mass ratio was 3:2, and the properties of both aggregates met the requirements of Chinese standard JGJ 52—2006.

**Figure 1:**
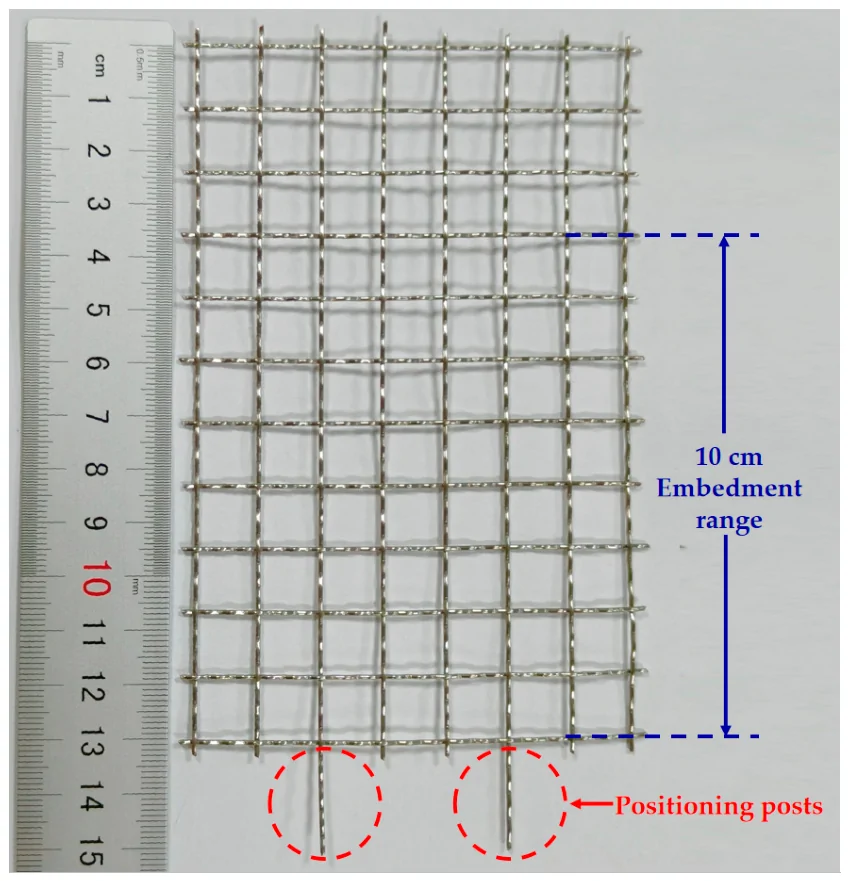
Structure and dimensions of the 316 stainless-steel wire mesh electrode.

Copper-coated straight steel fibers (CCSFs) were used as the conductive filler. Their geometric and mechanical properties are listed in Table 1. Type 316 stainless steel wire mesh was used as the electrodes for electrical resistivity measurements. The electrodes are shown in Figure 1, and their dimensions and properties are provided in Table 2.

**Figure 2:**
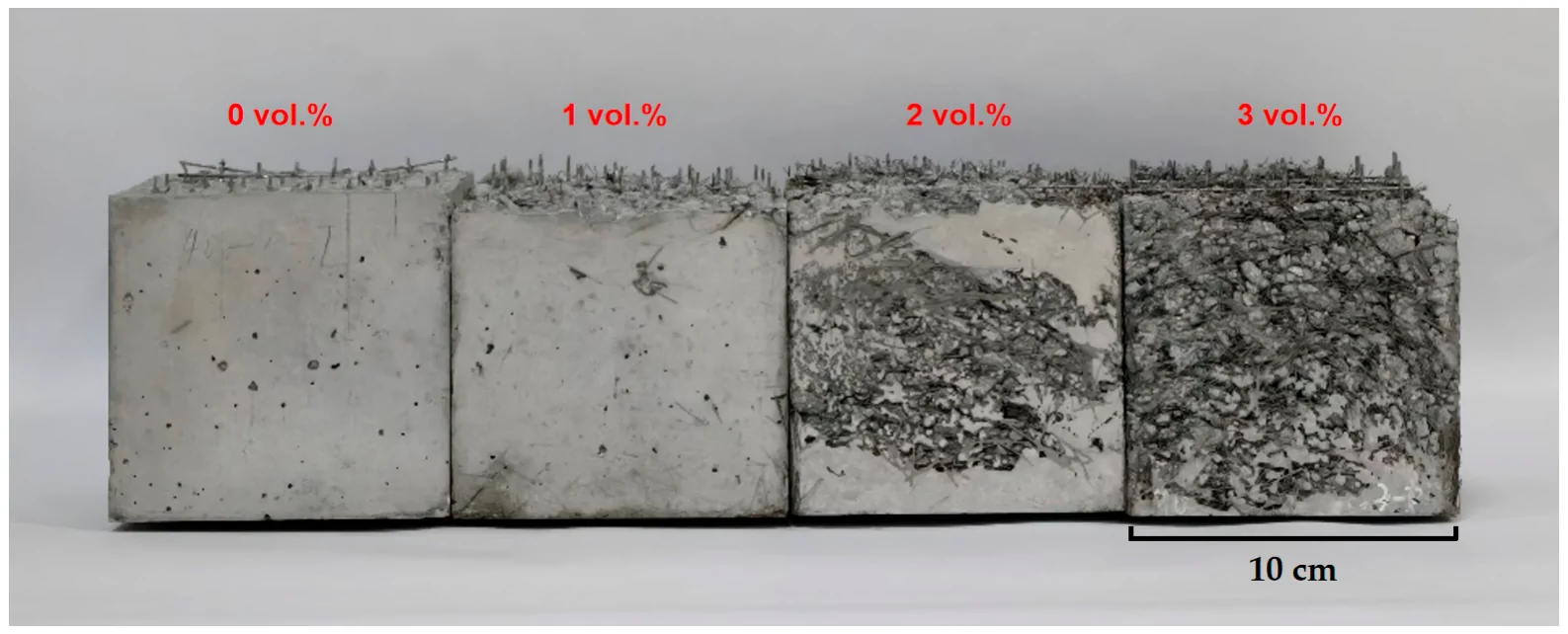
Appearance of specimens with different CCSF contents at a water-to-cement ratio of 0.40.

A series of preliminary mixture trials was conducted to ensure uniform dispersion of CCSF in the concrete and satisfactory specimen casting quality. The results showed that casting quality progressively deteriorated with increasing CCSF volume fraction. At a water-to-cement ratio of 0.40, visible casting defects, including honeycombing and surface voids, began to appear when the CCSF volume fraction reached 2% or higher, as shown in Figure 2. These observations indicated that high CCSF volume fractions hindered uniform fiber dispersion and adequate compaction at a low water-to-cement ratio. Increasing the water-to-cement ratio to 0.45 allowed specimens with CCSF volume fractions ranging from 0% to 3% to maintain satisfactory casting quality.

Based on the preliminary mixture optimization, the reference mixture was specified with mass proportions of cement:water:coarse aggregate:fine aggregate of 500:225:900:600. This corresponded to a water-to-cement ratio of 0.45 and a fine aggregate-to-total aggregate mass ratio of 0.40. The mixture proportions for all test groups are listed in Table 3.

### 2.2. Specimen Preparation Procedure

**Figure 3:**
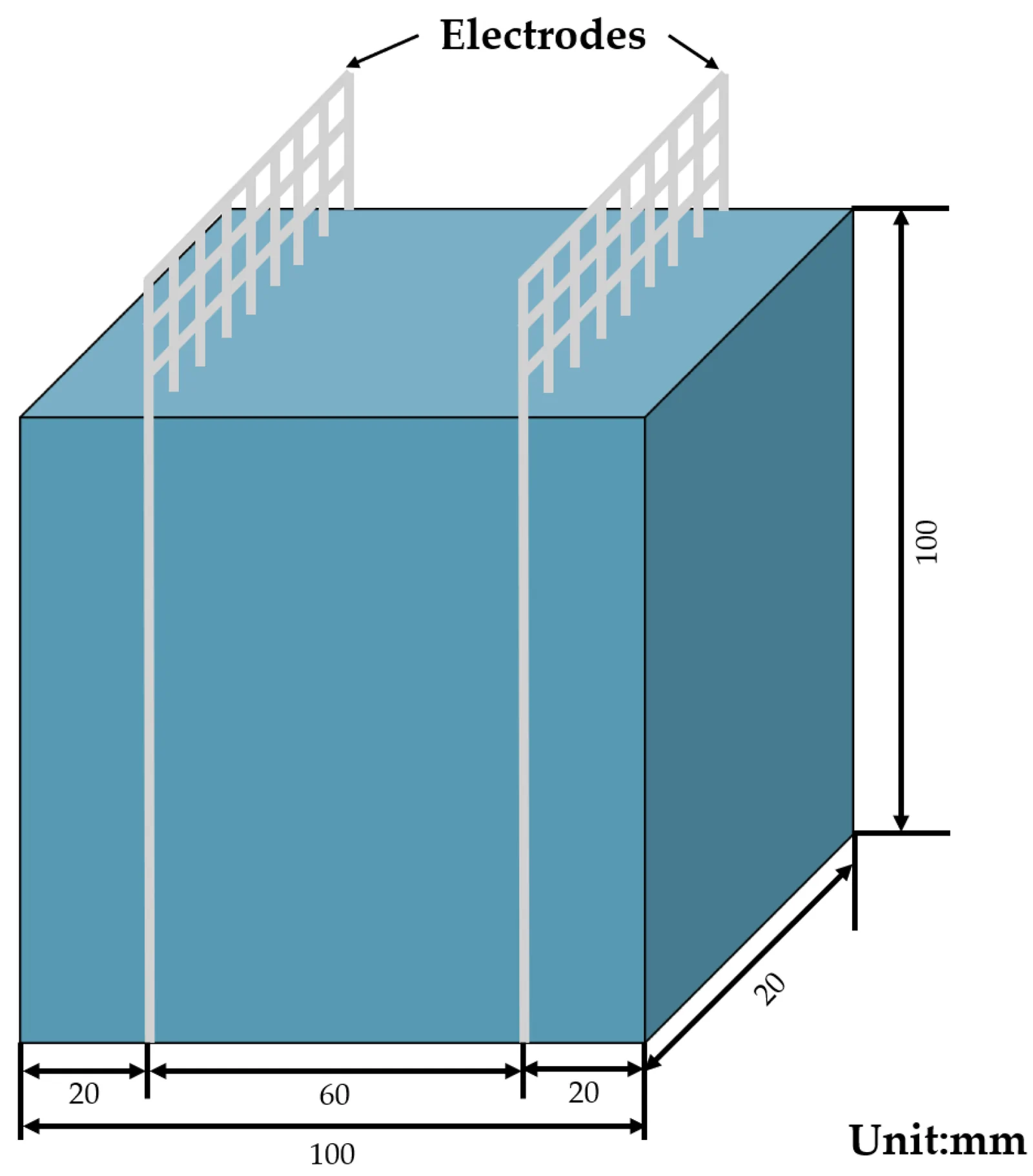
Schematic illustration of the electrode configuration in the specimen.

CCSF concrete specimens were prepared as cubes with a side length of 100 mm and an electrode spacing of 60 mm, as shown in Figure 3.

**Figure 4:**
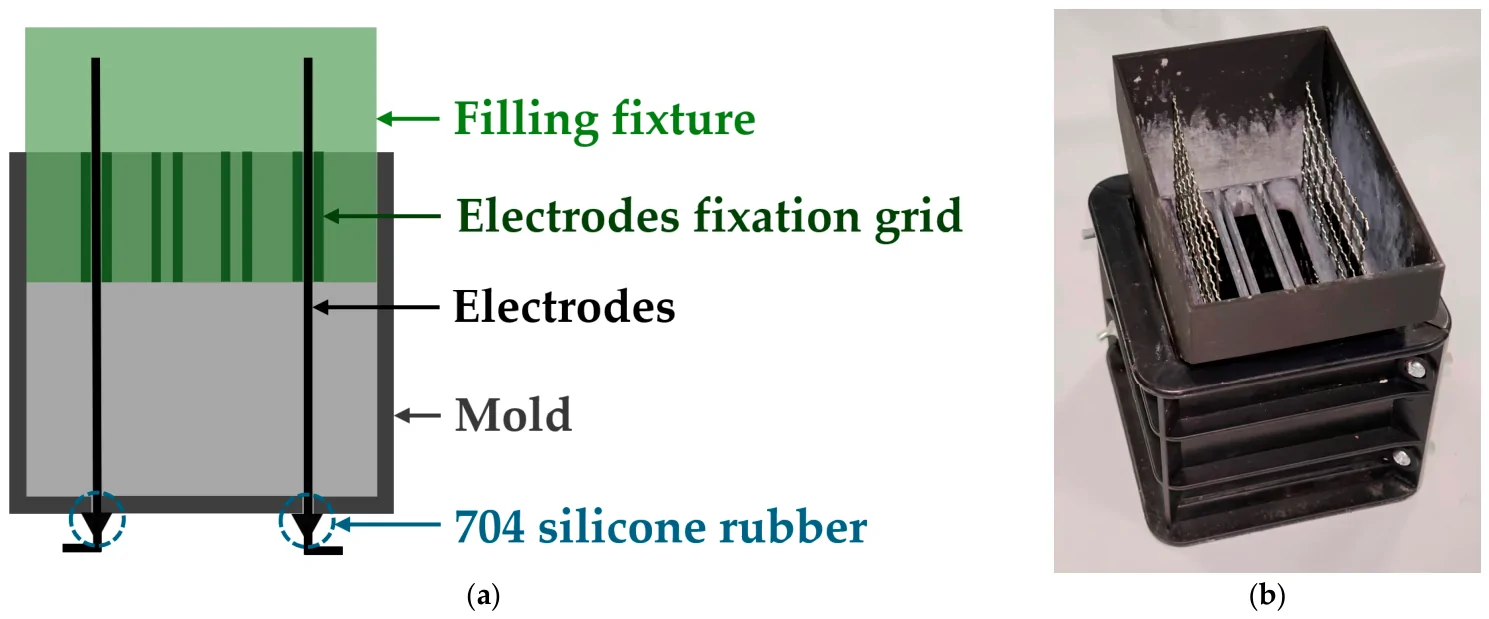
Electrode Fixation Method: (**a**) cross-sectional schematic. (**b**) photograph of the electrode fixation method.

**Figure 5:**
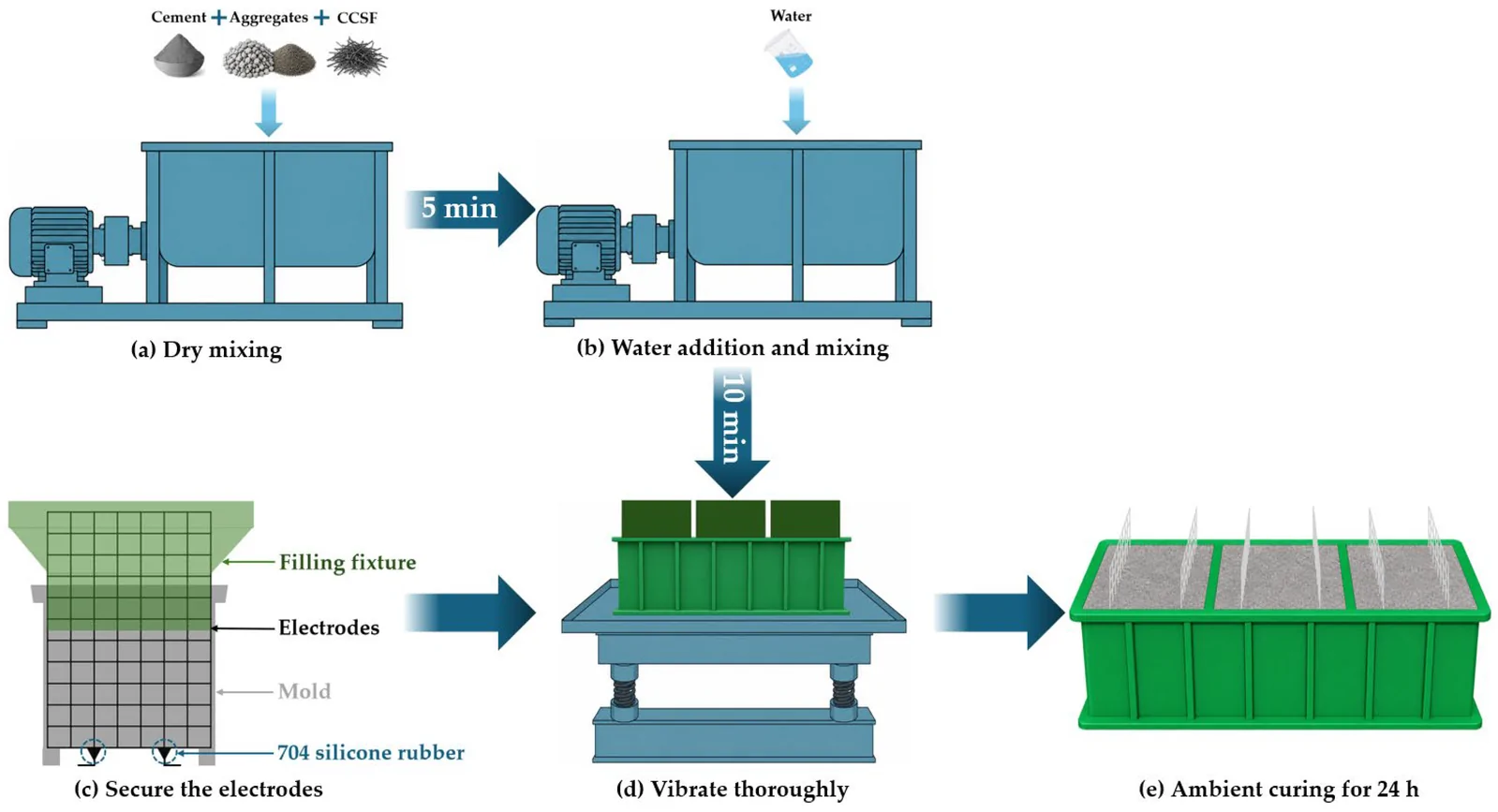
Flowchart of the specimen preparation procedure.

The electrode embedment procedure was modified to achieve more precise control of electrode spacing. In the modified procedure, positioning holes were first drilled in the molds using a 3D-printed positioning template. The positioning posts of the electrodes were then inserted into these holes. A 3D-printed feeding attachment incorporating an electrode fixation device was subsequently installed at the top of the mold, as shown in Figure 4. This attachment facilitated concrete placement and secured the upper ends of the electrodes to limit their displacement and rotation during casting and vibration. Type 704 silicone rubber was then applied at the bottom of the positioning holes to prevent leakage of cement slurry. Casting was performed after the silicone rubber had cured. The thoroughly mixed fresh concrete was then placed into the molds and compacted on a vibrating table. The exposed surfaces were covered with plastic film to prevent moisture evaporation. After curing under ambient conditions for 24 h, the specimens were demolded and transferred to a curing chamber (SHBY-60B, Xinmeihua Testing Instrument Co., Ltd., Hebei, China) maintained at 25 °C and 95% relative humidity. The specimen preparation procedure is illustrated in Figure 5.

In this study, a total of 249 specimens prepared using the modified procedure were used for compressive strength and electrical resistivity tests, and the consistency of electrode positioning was evaluated on these specimens. A vernier caliper was used to measure the clear spacing between the inner faces of the two electrodes at their left and right ends, denoted as d_1_ and d_2_, respectively. The relative spacing error and angular deviation were calculated using the following equations.

(1) $$\theta =\arctan(\frac{\left|d_{1}-d_{2}\right|}{L_{e}})$$

(2) $$\varepsilon_{d}=\frac{\left|\frac{d_{1}+d_{2}}{2}-L_{0}\right|}{L_{0}}\times 100\%$$

Here, *θ* is the deviation angle, *ε_d_* is the relative error, and *d*_1_ and *d*_2_ are the inner spacings at both ends of the electrode, respectively, *L*_e_ is the Electrode width (89 mm in this study), and *L*_0_ is the theoretical target spacing (6 cm in this study).

The mean absolute relative spacing error was 1.05%, with a maximum of 2.33%, while the mean angular deviation was 0.86°, with a maximum of 3.24°. The bottom ends of the electrodes were secured in predrilled holes and served as the positioning reference. Measurements showed that the bottom-end spacing remained essentially at the designed value with negligible displacement. Therefore, the bottom-end spacing was not included in the statistical analysis of positioning consistency, which focused on the top-end spacing that was more susceptible to displacement during casting and vibration. The same electrode-positioning procedure and operating sequence were followed for all specimens, supporting the repeatability of electrode placement under the preparation conditions used in this study.

**Figure 6:**
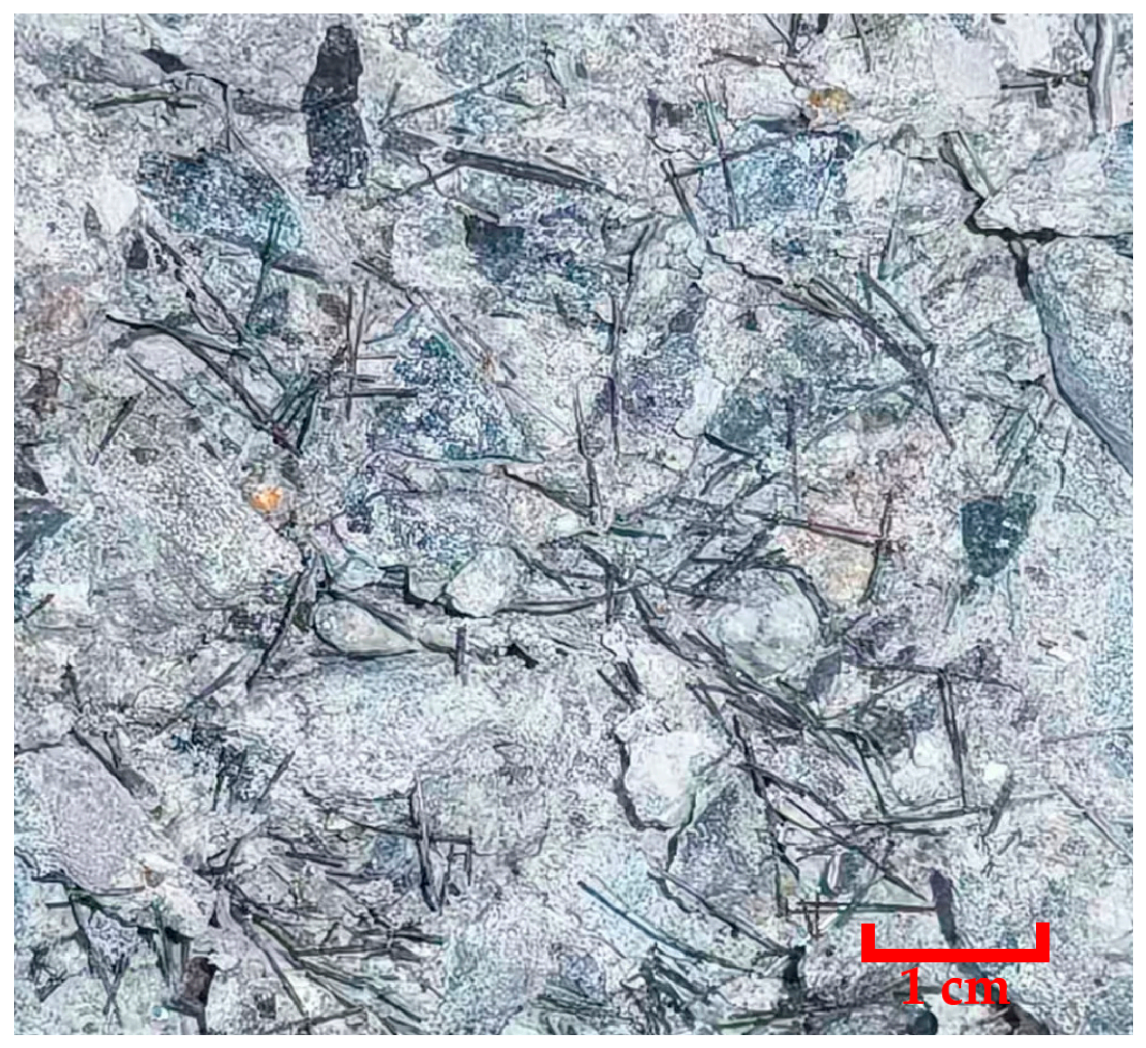
Cross-sectional view of the specimen.

The CCSFs were also distributed uniformly, as illustrated by the specimen cross-section in Figure 6. These results provided a basis for reducing experimental data scatter and improving the reliability of the measurements.

### 2.3. Measurement and Analysis Methods

#### 2.3.1. Unconfined Compressive Strength Test

The unconfined compressive strength of the CCSF concrete was determined using 100 mm cube specimens at an age of 28 d. A YAW6506 microcomputer-controlled electro-hydraulic servo compression testing machine (SANS, Shenzhen, China) was used. Force-controlled loading was applied at a rate of 3000 N/s until specimen failure. The compressive specimens were tested at room temperature. Three specimens were tested for each group, and the arithmetic mean with standard deviations was reported.

#### 2.3.2. Electrical Resistivity Measurement and Analysis Methods

The electrical behavior of CCSF concrete specimens is sensitive to measurement conditions. Factors such as specimen age, power supply type, electrode configuration, applied voltage, test specimen temperature and energization time can affect the measured electrical resistivity [3,4,17]. To ensure the accuracy and comparability of measurements across specimens, preliminary tests were conducted to compare direct current (DC) and alternating current (AC) power supplies, two-electrode and four-electrode methods, different specimen temperature and different applied voltages and specimen ages. A consistent measurement procedure and approach to controlling the test parameters were established based on these preliminary results.

**Figure 7:**
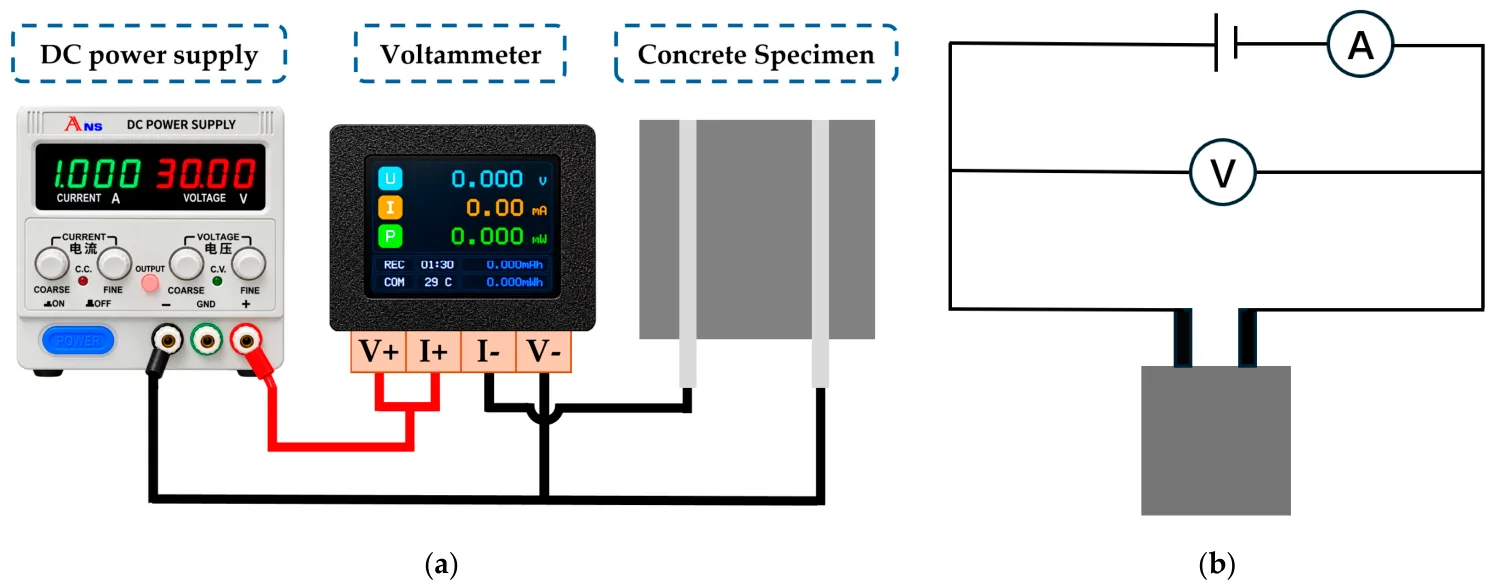
Schematic diagram of the testing circuit: (**a**) physical connection photograph. (**b**) equivalent circuit schematic.

The measurement circuit is shown in Figure 7. The electrical leads of the measurement circuit were connected to the exposed portions of the embedded 316 stainless steel wire mesh electrodes using alligator clips, thereby establishing electrical connections between the circuit and the specimen.

Electrical resistivity was used to characterize the static electrical properties of the concrete specimens and was calculated using Equation (3).

(3) $$\rho =\frac{\mathrm{RA}}{L}$$

Here, *ρ* is the electrical resistivity, *R* is the specimen resistance measured after 5 min of energization, *A* is the electrode area, and *L* is the electrode spacing. In this study, the effective electrode area was 89 cm^2^, and the electrode spacing was 60 mm.

However, electrical resistivity describes the electrical behavior of a material only under specified conditions and does not directly quantify its response to changes in an influencing factor. *A* resistivity sensitivity coefficient, *S*, was therefore introduced to evaluate the sensitivity of concrete resistivity to changes in each factor over the interval between adjacent factor levels, as expressed in Equation (4).

(4) $$S_{\rho }=-\frac{\Delta \rho }{\Delta V_{f}}=-\frac{\rho_{i}-\rho_{i-1}}{V_{i}-V_{i-1}}$$

Here, *ρ*_i_ and *ρ*_i−1_ are the electrical resistivities at the ith and (i − 1)th factor levels, respectively, and the corresponding factor levels are the values of the factor being investigated. *A* larger *S* indicates a greater decrease in resistivity per unit change in the factor over the specified interval and thus a greater sensitivity to that factor.

For each test condition, three independently prepared specimens were tested. The electrical resistivity of each specimen was calculated using Equation (3), and the reported resistivity values are presented as arithmetic means ± standard deviations. The error bars in the resistivity figures represent the standard deviations among these three independent specimens, rather than repeated measurements on a single specimen. The sensitivity coefficient S was calculated from the mean resistivity values at adjacent factor levels.

Compared with AC measurements, DC measurements directly capture the current response of the material under a constant applied electric field. The measurement system requires only conventional equipment, such as a DC power supply, an ammeter, and a voltmeter, without the more complex and expensive equipment associated with AC characterization, such as an impedance analyzer or a lock-in amplifier. This makes the DC method more suitable for practical implementation. However, DC measurements must account for polarization effects arising from ion migration and interfacial charge accumulation within the specimen. During the initial stage of energization, the measured resistivity increased considerably with time and began to stabilize after approximately 2 min. The resistivity measured at 10 min was only 1.47% higher than that measured at 5 min, indicating that the polarization response had largely stabilized. Accordingly, a DC power supply was used in this study, and resistivity was calculated from the current and voltage measured after 5 min of energization.

Comparison of the preliminary results obtained using the two-electrode and four-electrode methods showed that, in the four-electrode configuration examined, the voltage applied between the outer electrodes was not uniformly distributed across the specimen. The voltage drop across the central measurement section was considerably greater than those across the two outer sections. In the four-electrode configuration, the non-measurement sections also shared part of the applied voltage; thus, the voltage across the measurement section was only a fraction of the total applied voltage. Reaching the target measurement-section voltage therefore required a higher total external voltage, which placed stricter demands on the power supply and increased Joule heating in the specimen, potentially disturbing the resistivity measurement. The DC two-electrode method was therefore adopted to measure the electrical resistivity of the concrete specimens.

The preliminary tests also showed that the electrical resistivity of CCSF concrete increased with specimen age, while the rate of increase gradually declined. To balance experimental efficiency and repeatability, all measurements were conducted on specimens at an age of 28 days.

The electrical resistivity of CCSF concrete exhibited a pronounced negative correlation with the voltage applied across the specimen. At low applied voltages, the specimens had high resistivity and carried small currents, resulting in weak electrical signals. Under these conditions, the measurements were more susceptible to voltage fluctuations, electrode contact conditions, and instrument resolution, placing greater demands on measurement accuracy. As the applied voltage increased, the measured signals became stronger, the relative errors caused by external disturbances decreased, and the resistivity measurements became substantially more stable. Nevertheless, a higher applied voltage is not necessarily preferable. The Joule heat generated in a specimen during energization can be expressed as follows:

*Q = I*^2^*Rt* (5)

Here, *Q* is the heat generated in the specimen, *I* is the current passing through it, *R* is the specimen resistance, and *t* is the energization time.

**Figure 8:**
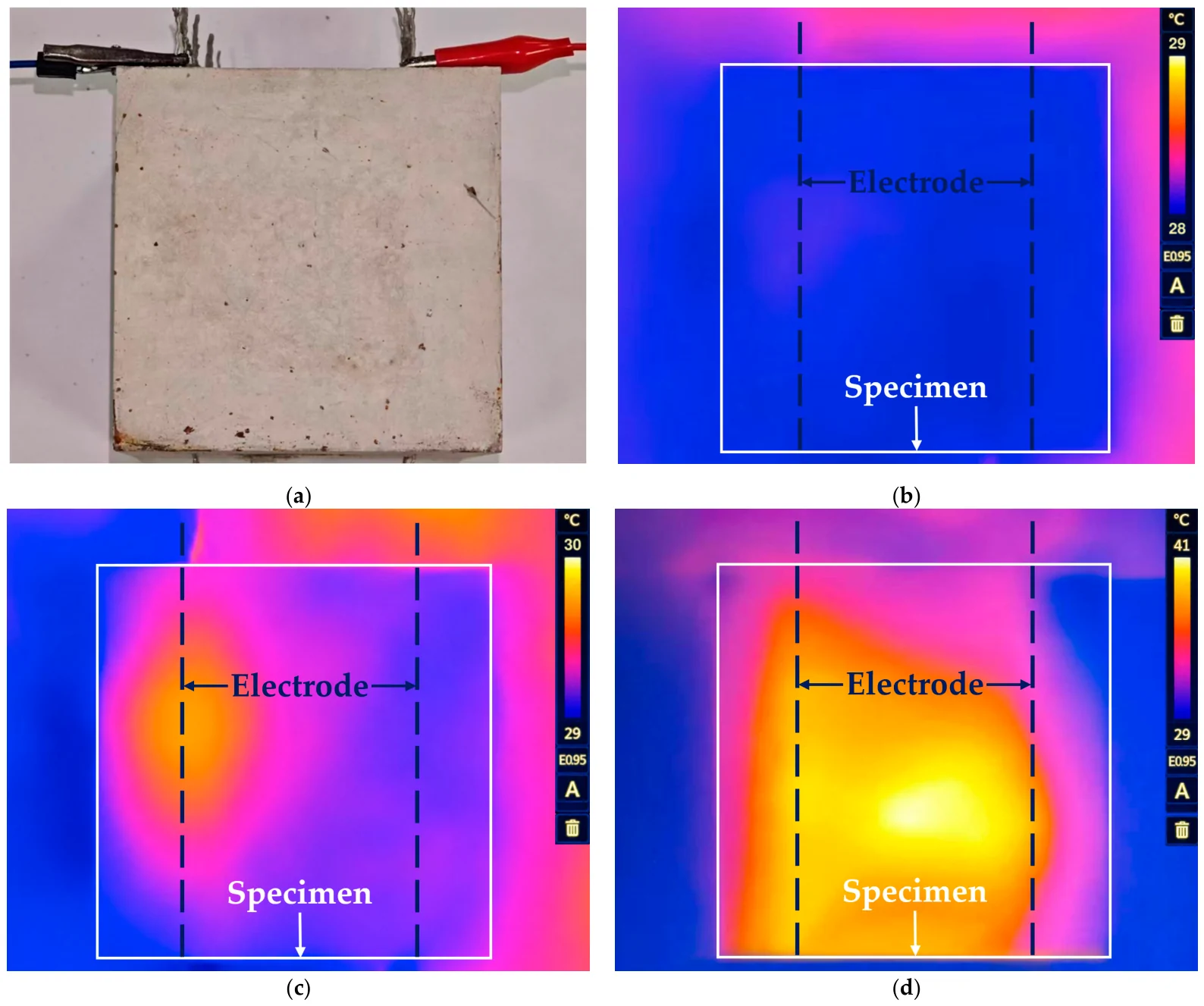
Infrared thermographic images showing Joule heating in the specimen (CCSF volume fraction = 3.0%; water-to-cement ratio (W/C) = 0.45; equilibrated outdoors at approximately 28 °C for 48 h prior to testing): (**a**) In-situ physical image of the specimen. (**b**) Infrared thermographic image before applying voltage. (**c**) Infrared thermographic image under an applied voltage of 30 V for 5 min. (**d**) Infrared thermographic image under an applied voltage of 60 V for 5 min.

Increasing the applied voltage increased the current through the specimen and substantially increased Joule heating [7,23]. The resulting temperature rise could, in turn, affect the measured resistivity. Figure 8 presents infrared thermographic images of the specimen before energization and after 5 min of energization at applied voltages of 30 V and 60 V, together with a photograph of the same specimen region monitored by the infrared camera (Testo 875 Pro, Titisee-Neustadt, Germany). Compared with the initial condition, only a slight increase in surface temperature was observed at 30 V, whereas appreciable heating occurred at 60 V. The temperature rise at 30 V was considered negligible under the tested conditions. Accordingly, 30 V was selected as the reference applied volt-age to balance measurement signal stability against specimen heating.

Because specimen temperature also affects the measured electrical resistivity, it was controlled throughout testing. For resistivity tests in which temperature was not a test variable, specimens were removed from the curing chamber maintained at 25 °C and tested immediately. In the variable-temperature tests, specimens were sealed with plastic film to minimize changes in moisture content during temperature variations and placed in a high–low temperature humidity chamber. After the specimens reached each target temperature, they were maintained at that temperature for an additional 48 h to allow thermal equilibration before electrical measurements. The surface temperature of each specimen was monitored using an infrared thermal imaging camera (Testo 875 Pro, Germany) (emissivity set to 0.95), with the ambient temperature during testing recorded as 26–29 °C. The camera was factory-calibrated, and its measurement error was verified using a reference object at a known temperature and found to be within the tolerance required for the present experiments. The measured temperature was taken as the average temperature within a rectangular region corresponding to the 60 mm test section between the electrodes.

Accordingly, the DC two-electrode method was adopted to minimize the influence of the measurement method and test conditions on the measured electrical resistivity of CCSF concrete. The reference test conditions comprised an applied voltage of 30 V, a polarization time of 5 min, and a specimen age of 28 days, providing a consistent basis for accurate and comparable measurements when individual test variables were varied.

## 3. Results and Discussion

### 3.1. Effect of CCSF Volume Fraction on the Mechanical Properties of Concrete

**Figure 9:**
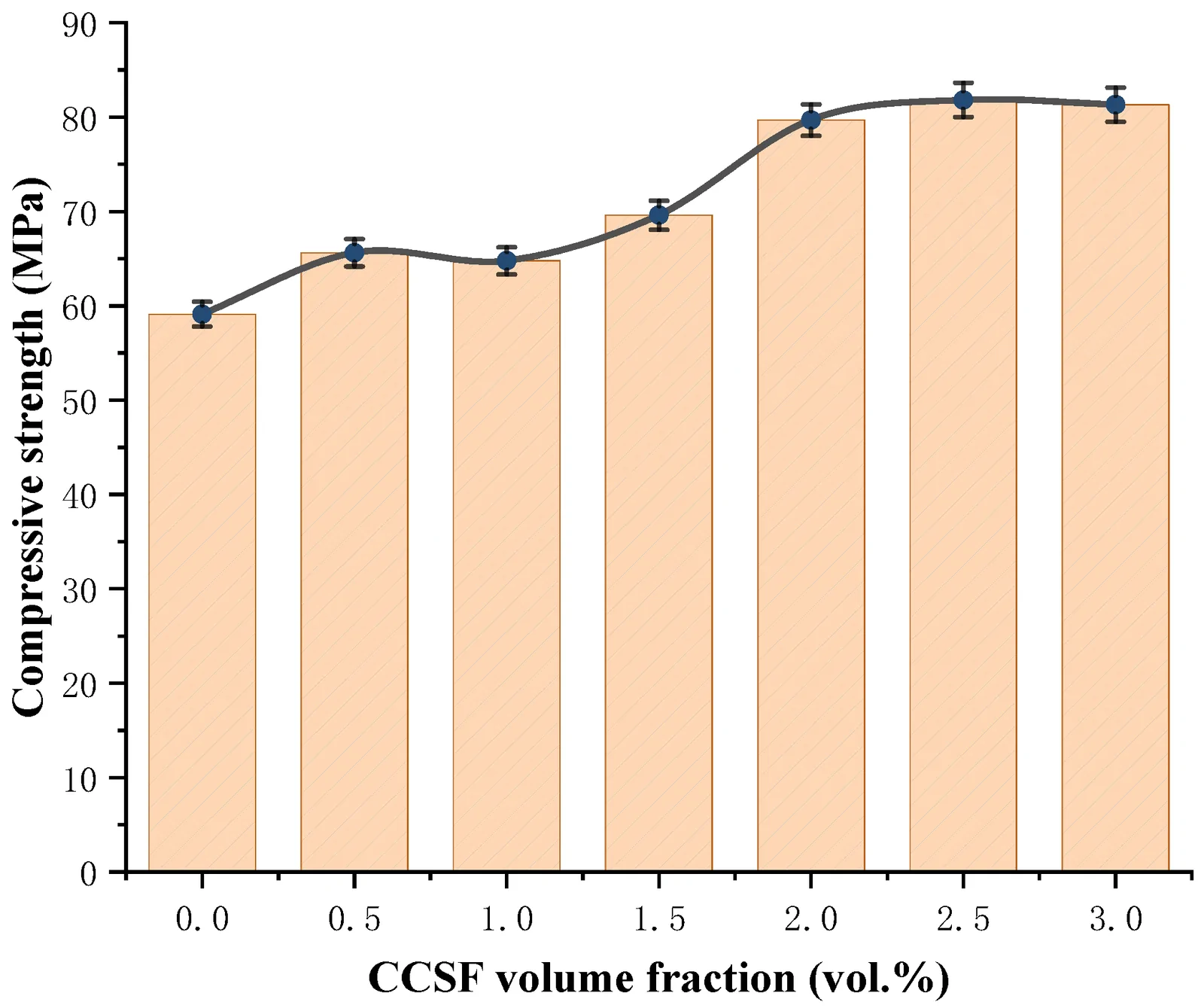
Compressive strength curves of concrete specimens with different CCSF volume fractions (W/C = 0.45).

To assess the suitability of CCSF concrete for engineering applications, unconfined compressive strength tests were conducted on specimens with different CCSF volume fractions. The results are shown in Figure 9. The compressive strength generally increased with increasing CCSF volume fraction, indicating that the incorporation of CCSF did not compromise the load-bearing capacity of the concrete. Within the range of CCSF volume fractions investigated, all specimen groups exhibited high compressive strength, meeting the requirements for subsequent electrical performance tests and relevant engineering applications.

### 3.2. Effect of CCSF Volume Fraction on the Electrical Properties of Concrete

**Figure 10:**
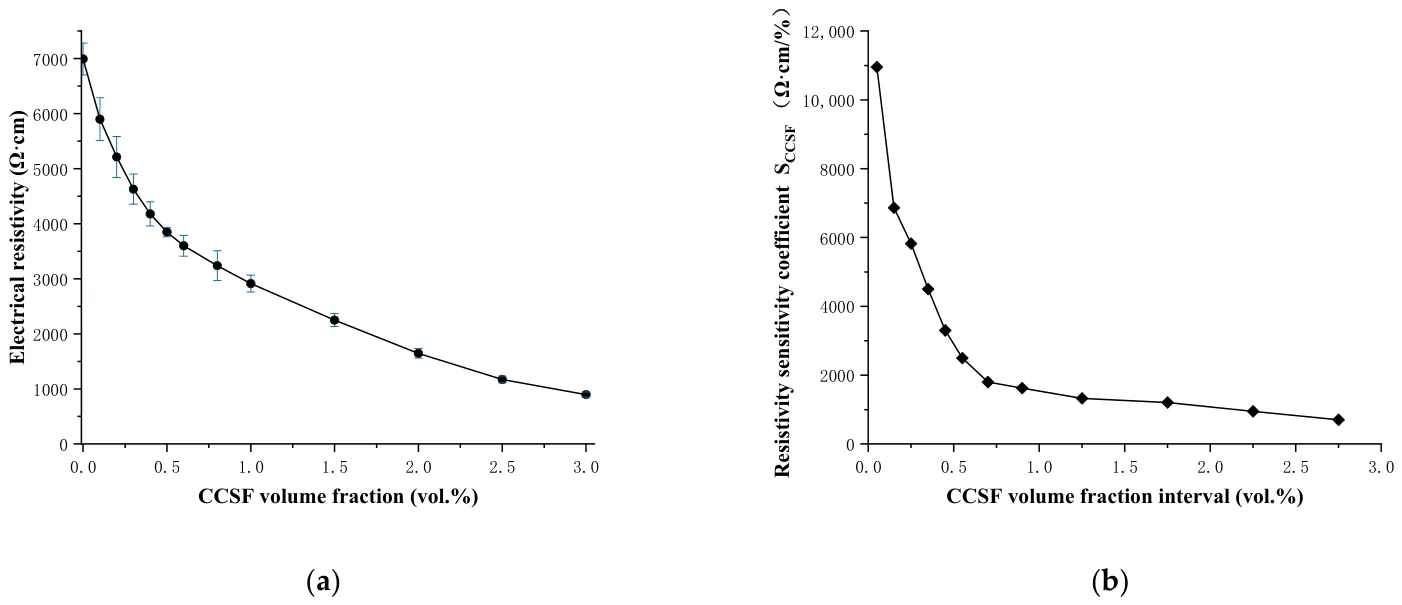
Effect of CCSF volume fraction on the electrical properties of concrete (W/C = 0.45 and an applied voltage of 30 V): (**a**) Relationship between specimen electrical resistivity and CCSF volume fraction, *p* = 2.382 × 10^−22^. (**b**) Relationship between the resistivity sensitivity coefficient S_CCSF_ and CCSF volume fraction.

The experimental results showing the effect of CCSF volume fraction on the electrical resistivity of concrete are presented in Figure 10a. The resistivity decreased continuously with increasing CCSF volume fraction. As the CCSF volume fraction increased from 0% to 3.0%, the resistivity decreased from 6993.46 Ω·cm to 896.78 Ω·cm, representing an overall reduction of 87.18%. This indicates that incorporating CCSF substantially improved the electrical conductivity of concrete. However, the decrease in resistivity became progressively smaller as the fiber volume fraction increased, indicating that the electrical response to changes in CCSF volume fraction differed considerably across the investigated intervals.

The sensitivity coefficient of electrical resistivity with respect to CCSF volume fraction, S_CCSF_, calculated using Equation (4), is shown in Figure 10b. Overall, the resistivity sensitivity coefficient S_CCSF_ decreased with increasing CCSF volume fraction. In the 0–0.1% interval, S_CCSF_ reached 10,954.38 Ω·cm/%, indicating that even a small addition of CCSF caused a substantial reduction in resistivity and that the electrical properties were highly sensitive to changes in CCSF volume fraction. Thereafter, S_CCSF_ decreased rapidly, with a pronounced change in trend around the 0.6–0.8% interval. Above 0.8%, its variation became more gradual, the decrease over the higher-fiber-content interval accounts for 12.02% of the total decrease over the investigated range. Based on the variations in resistivity and S_CCSF_, the effect of CCSF on the electrical properties of concrete can be divided into two regimes: 0–0.8% and 0.8–3.0%.

In the low fiber volume fraction regime, increasing the CCSF volume fraction from 0% to 0.8% reduced the electrical resistivity from 6993.46 Ω·cm to 3239.19 Ω·cm, corresponding to a reduction of 53.68%. Meanwhile, S_CCSF_ decreased rapidly from 10,954.38 to 1801.21 Ω·cm/%, representing a reduction of 83.56%. The pronounced decrease in resistivity and the high values of S_CCSF_ indicate that the electrical properties were particularly sensitive to changes in CCSF volume fraction within this regime. In the absence of CCSF, concrete lacks effective electron transport pathways, and charge transport relies primarily on ion migration through the pore solution, resulting in high resistivity. When a small amount of CCSF is incorporated, the fibers can bridge locally conductive regions and shorten charge transport distances, progressively connecting previously isolated transport pathways. As the CCSF volume fraction increases further, the mean distance between fibers decreases, increasing the likelihood of direct contact or charge transport over short distances. The number of effective conductive pathways therefore increases rapidly, leading to a substantial decrease in resistivity. Meanwhile, as conductive pathways gradually form, the additional reduction in resistivity per unit increase in fiber volume fraction begins to diminish, resulting in a rapid decline in S_CCSF_.

When the CCSF volume fraction exceeds 0.8%, the system enters the high fiber volume fraction regime.

Increasing the CCSF volume fraction from 0.8% to 3.0% further reduced the resistivity from 3239.19 Ω·cm to 896.78 Ω·cm, the decrease over the 0.8–3.0% fiber-content interval accounts for 38.42% of the total decrease over the 0–3.0% range. Meanwhile, SCCSF decreased from 1801.21 to 548.82 Ω·cm/%, the decrease over the higher-fiber-content interval accounts for 12.02% of the total decrease over the investigated range.

These results indicate that, above a CCSF volume fraction of 0.8%, the reduction in resistivity per unit increase in fiber volume fraction changed relatively little, and the sensitivity to CCSF volume fraction tended to stabilize. This behavior can be attributed to the presence of numerous conductive pathways with a high degree of connectivity within the specimens. Additional CCSFs primarily supplement and improve the existing conductive network by enhancing local connectivity and reducing contact resistance between fibers, rather than establishing new continuous transport pathways. As the role of additional CCSF gradually shifts from forming pathways to improving existing ones, their contribution to further reductions in overall resistivity becomes relatively limited. Consequently, S_CCSF_ decreases only slightly and gradually stabilizes.

**Figure 11:**
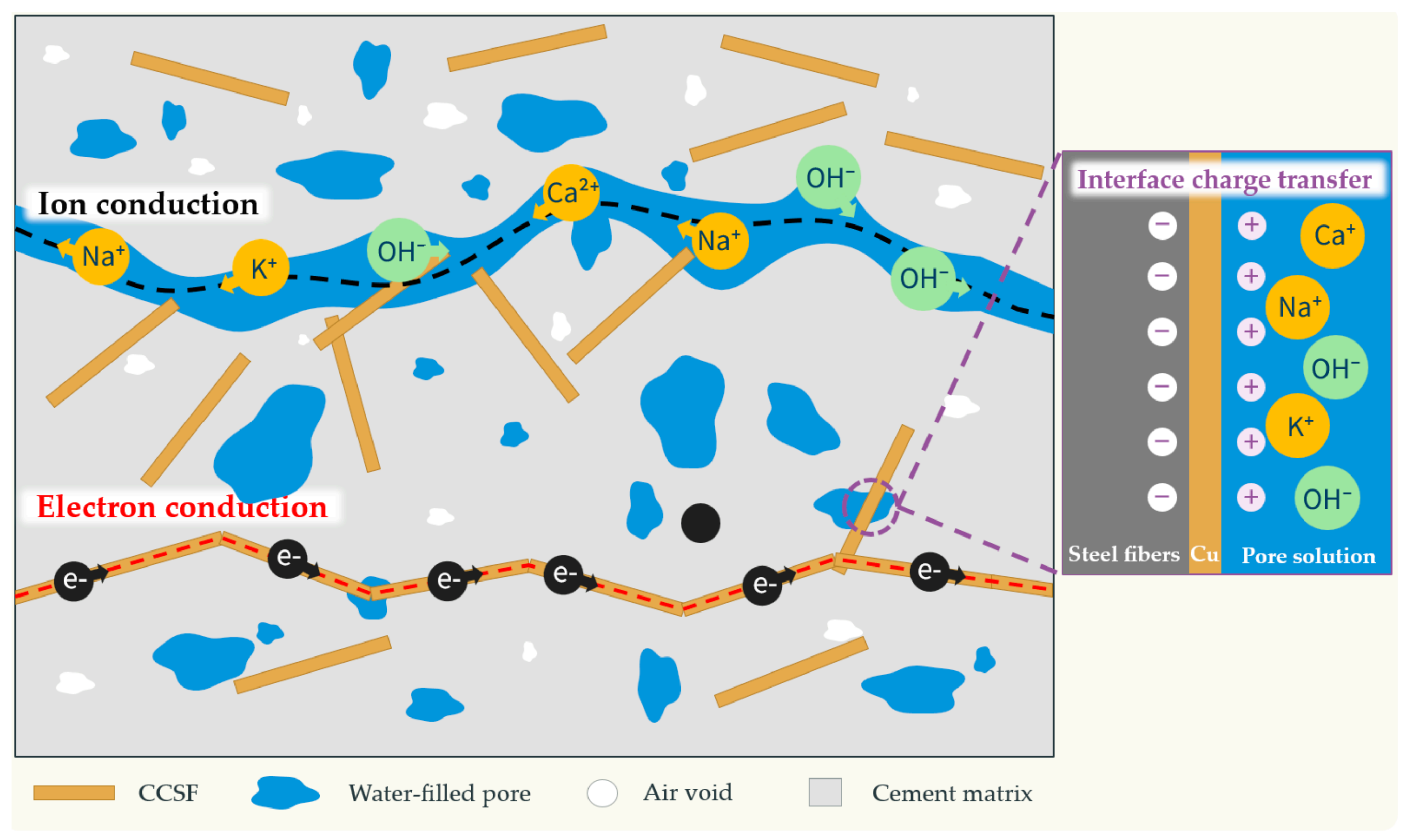
Schematic illustration of the charge-transport pathways in CCSF concrete.

Overall, charge transport in CCSF concrete occurs through three principal pathways. As illustrated in Figure 11, ionic conduction through the pore solution, electronic conduction through the CCSF network, and interfacial charge transfer between the CCSF and the surrounding cementitious matrix. In the low fiber volume fraction regime of 0–0.8%, conductive pathways develop rapidly, and resistivity is highly sensitive to changes in CCSF volume fraction. Above 0.8%, conductive pathways are largely established, and additional CCSFs primarily improve the connectivity and stability of the existing network. Resistivity therefore continues to decrease, while changes in S_CCSF_ become substantially smaller. These findings indicate that the improvement in the electrical conductivity of concrete depends not only on the amount of conductive material incorporated but also on how the CCSFs modify the internal charge transport pathways and conductive network [13]. It should be emphasized that 0.8% is an empirical response-regime boundary identified from the changes in resistivity and *S*_CCSF_, rather than a quantitatively determined percolation threshold.

### 3.3. Effect of Water-to-Cement Ratio on the Electrical Properties of Concrete

**Figure 12:**
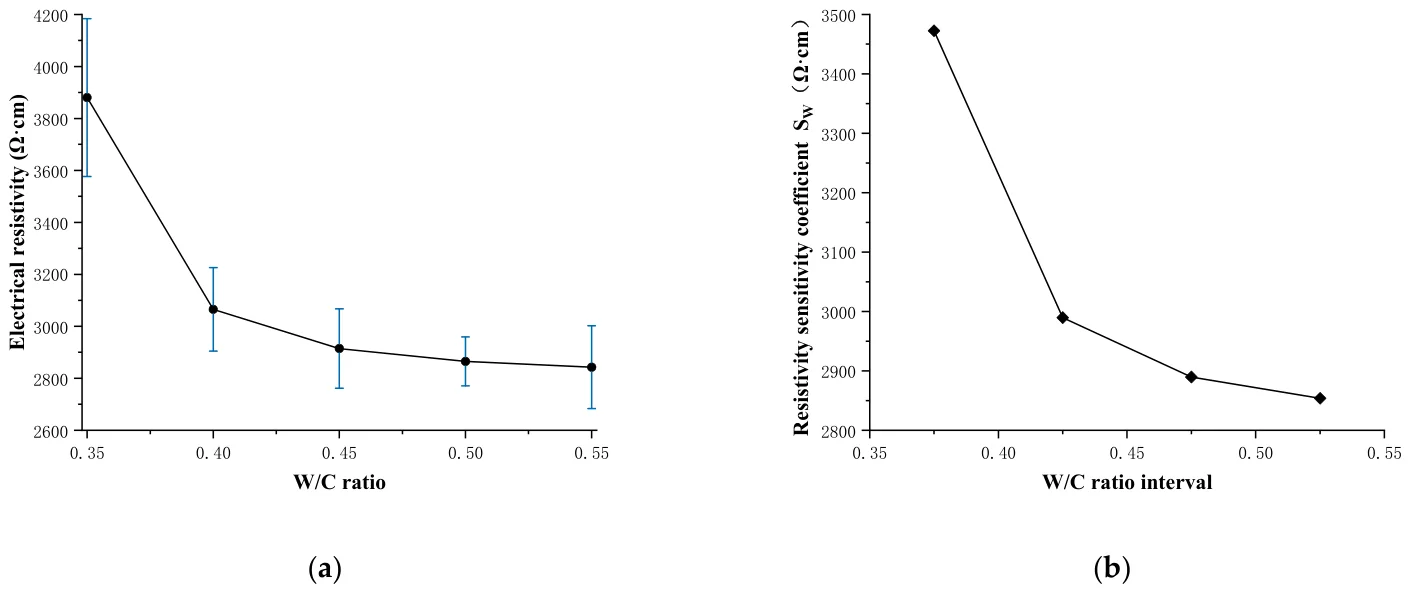
Effect of water-to-cement ratio on the electrical properties of concrete (CCSF volume fraction = 1.0% and an applied voltage of 30 V): (**a**) Relationship between specimen electrical resistivity and water-to-cement ratio, *p* = 0.00022. (**b**) Relationship between the resistivity sensitivity coefficient S_W_ and water-to-cement ratio.

The experimental results showing the effect of the water-to-cement ratio on the electrical resistivity of concrete are presented in Figure 12a. The resistivity gradually decreased as the water-to-cement ratio increased. Increasing the water-to-cement ratio from 0.35 to 0.55 reduced the resistivity from 3880.36 Ω·cm to 2842.72 Ω·cm, corresponding to a reduction of 26.74%. This indicates that increasing the water-to-cement ratio can improve electrical conductivity to some extent. Most of the change in resistivity occurred within the lower water-to-cement ratio range, followed by a progressively smaller decrease, revealing two distinct regimes.

The sensitivity coefficient of electrical resistivity with respect to the water-to-cement ratio, S_W_, is shown in Figure 12b. The coefficient decreased continuously with increasing water-to-cement ratio. Within the 0.35–0.40 interval, S_W_ was 16,297 Ω·cm, considerably higher than the values in the other intervals. Based on the variations in resistivity and S_W_, the effect of the water-to-cement ratio can be divided into two regimes: 0.35–0.40 and 0.40–0.55.

At water-to-cement ratios of 0.35 and 0.40, the resistivity values were 3880.36 Ω·cm and 3065.51 Ω·cm, respectively, corresponding to a reduction of 21.00%. This indicates that the electrical properties were most sensitive to changes in the water-to-cement ratio within this interval. At a low water-to-cement ratio, the relatively low flowability and compactability of the fresh mixture can result in internal voids and locally poorly compacted regions, which reduce the effective cross-sectional area available for electrical conduction and increase the tortuosity of charge transport pathways. Meanwhile, the relatively low pore solution content and limited continuity of the pore solution restrict ion migration and charge transfer between CCSFs that are not in direct contact. Increasing the water-to-cement ratio to 0.40 substantially improves matrix compactness and pore solution continuity, reducing resistance to ion migration and local interfacial charge transport and thereby causing a rapid decrease in resistivity [27].

The decrease in resistivity over the water-to-cement ratio interval of 0.40–0.55 accounted for 21.48% of the total decrease over the investigated range of 0.35–0.55, while S_W_ rapidly declined to a low level, indicating a substantially weaker influence of the water-to-cement ratio. In this regime, the internal structure of the concrete is relatively well developed, and further increases in the water-to-cement ratio provide only limited improvements in the effective cross-sectional area available for conduction and the continuity of transport pathways. Because the CCSF volume fraction remains constant, the network of electronic conduction pathways remains largely unchanged. Increasing the water-to-cement ratio therefore acts primarily by increasing the pore solution content and reducing resistance to ion migration. In particular, above a water-to-cement ratio of 0.45, the resistivity decreased by only 1.85%. This suggests that the water-to-cement ratio primarily enhances the supplementary contribution of ionic conduction at this stage, with limited influence on the overall electrical behavior governed by the CCSF.

Overall, once the water-to-cement ratio provides sufficient flowability for casting and allows adequate compaction, its influence on resistivity becomes limited. Increasing the water-to-cement ratio primarily reduces resistivity by enhancing ionic conduction through the pore solution. In the present tests, the CCSF volume fraction was fixed at 1%, and electronic conduction dominated charge transport, with a limited contribution from ionic conduction. Consequently, further increases in the water-to-cement ratio had little effect on electrical resistivity.

### 3.4. Effect of Temperature on the Electrical Properties of Concrete

**Figure 13:**
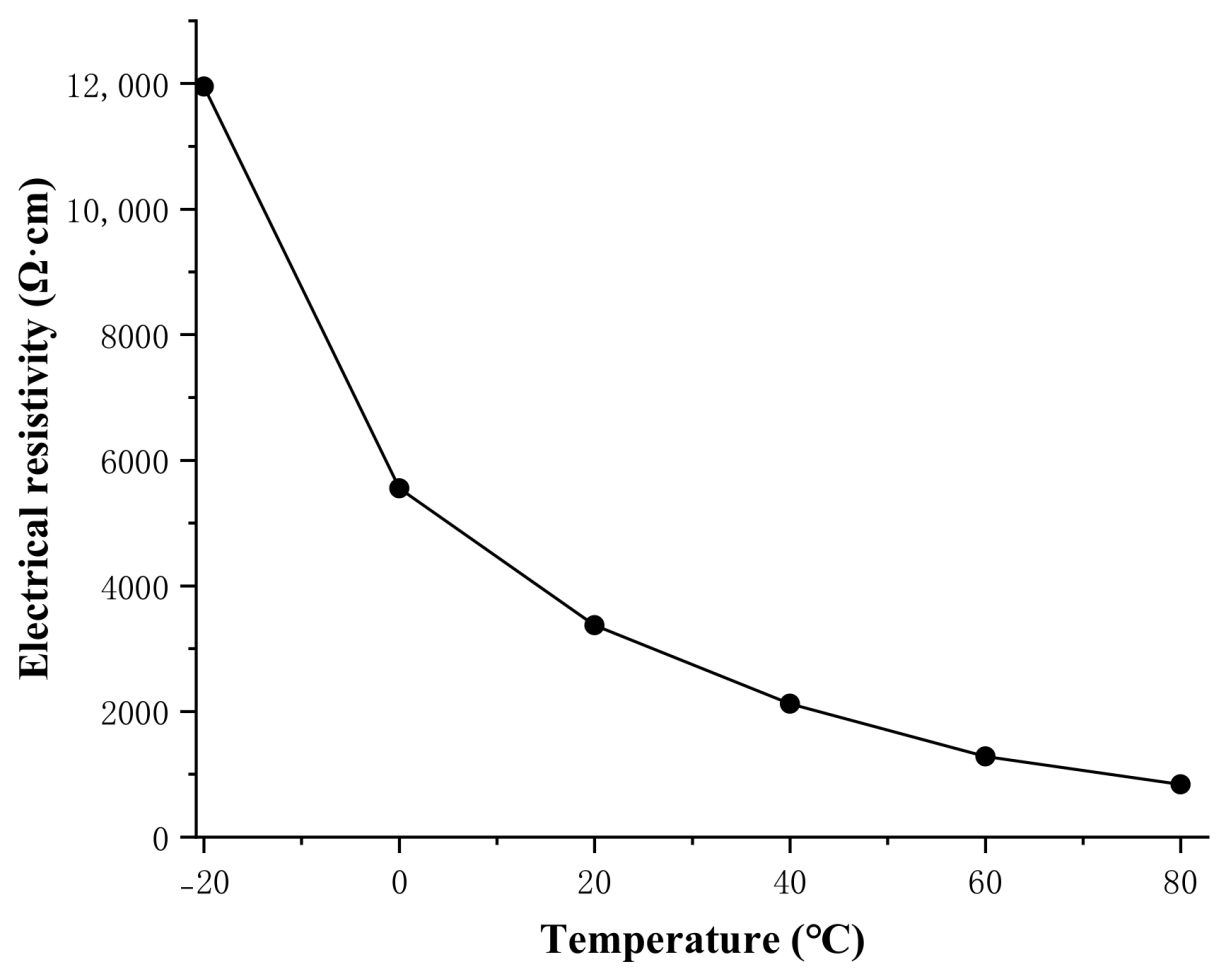
Relationship between specimen electrical resistivity and temperature, *p* = 1.973 × 10^−9^ (W/C = 0.40, CCSF volume fraction = 1.0%, and an applied voltage of 30 V).

The experimental results showing the effect of temperature on the electrical resistivity of concrete are presented in Figure 13. For specimens with a CCSF volume fraction of 1% and a water-to-cement ratio of 0.40, resistivity decreased continuously with increasing temperature. As the temperature increased from −20 °C to 80 °C, the resistivity decreased from 11,952.73 Ω·cm to 837.40 Ω·cm, corresponding to an overall reduction of 92.99%. The reduction within the −20–0 °C interval was 53.53%, followed by progressively smaller decreases over subsequent temperature intervals, indicating a pronounced nonlinear decay. Fitting the experimental data over the temperature range of 0 to 80 °C yielded the relationship given in Equation (6).

*ρ* = 115.09 + 5435.45e^−0.025285*T*^ (6)

where *T* is the temperature in °C and *ρ* is the electrical resistivity in Ω·cm. The coefficient of determination was *R*^2^ = 0.9999, indicating that the model described the temperature dependence of resistivity well within the investigated range.

The exponential decrease in resistivity with increasing temperature indicates that the temperature response is not governed by the metallic conduction characteristics of the CCSF alone but is closely associated with changes in the overall conductive pathways within the composite. Although CCSFs provide high electronic conductivity and can form local pathways of low electrical resistance within the cementitious matrix, their relatively large size means that the number of fibers is limited at a volume fraction of 1%. Their random distribution also makes it difficult to establish a fully continuous metallic conductive network. Consequently, current must still pass through the cementitious matrix between fibers. When conductive pore solution is present, increasing temperature promotes ion migration and reduces resistance to charge transport. This facilitates charge transfer between discontinuous fiber-based conductive pathways [28], leading to a continuous decrease in the overall resistivity of the specimens.

**Figure 14:**
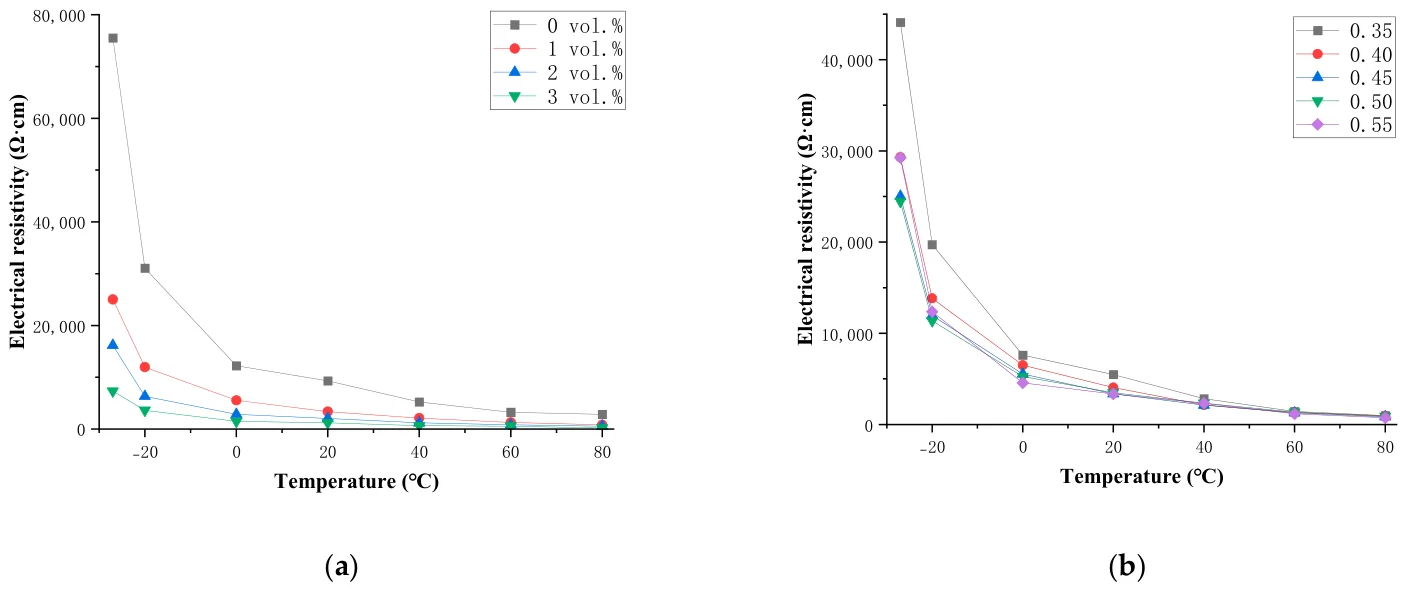
Electrical resistivity as a function of temperature for different mixture proportions (An applied voltage of 30 V): (**a**) Relationship between specimen electrical resistivity and temperature at different CCSF volume fractions (W/C = 0.45). (**b**) Relationship between specimen electrical resistivity and temperature at different water-to-cement ratios (the CCSF volume fraction was kept at 1.0%).

To further investigate the influence of mixture proportions on the temperature response, the resistivities of specimens with different CCSF volume fractions and water-to-cement ratios were measured at different temperatures, as shown in Figure 14. All groups exhibited a continuous decrease in resistivity with increasing temperature, consistent with the results in Figure 13. However, the magnitude of the decrease was closely associated with the absolute resistivity values of each group.

**Figure 15:**
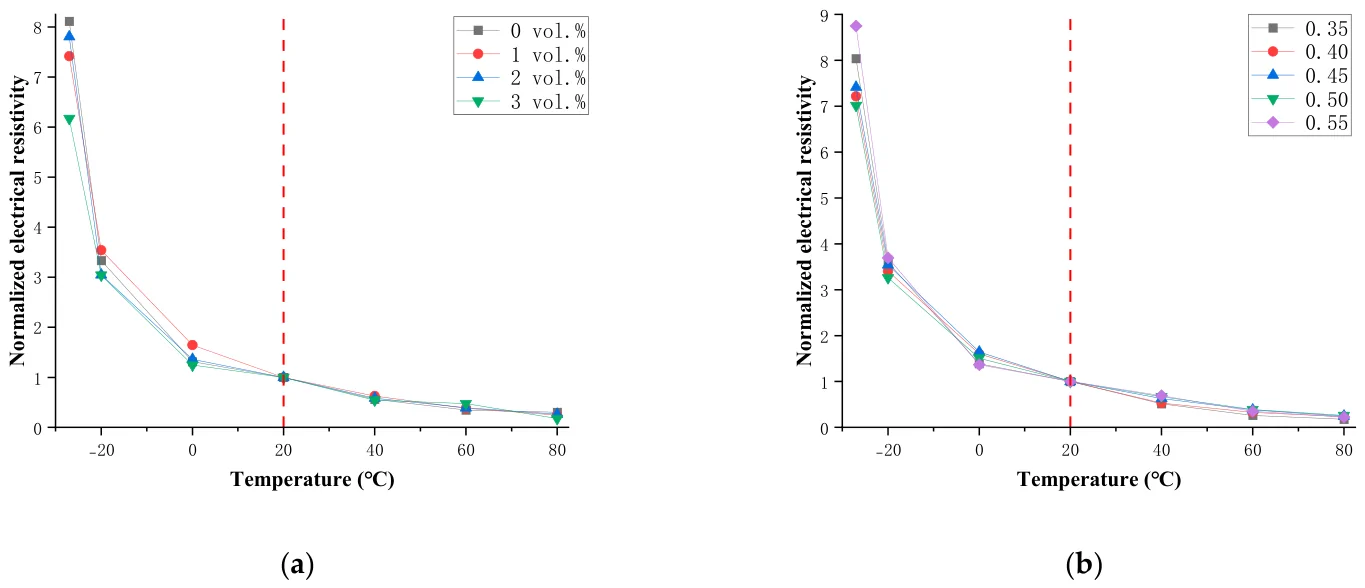
Normalized electrical resistivity as a function of temperature for different mixture proportions (an applied voltage of 30 V): (**a**) Relationship between normalized electrical resistivity and temperature at different CCSF volume fractions (W/C = 0.45). (**b**) Relationship between normalized electrical resistivity and temperature at different water-to-cement ratios(CCSF volume fraction = 1.0%).

To account for differences in absolute resistivity among specimens, the resistivity data for each specimen were normalized by its reference resistivity at 20 °C. As shown in Figure 15, the temperature dependence of normalized resistivity was broadly similar across specimens with different CCSF volume fractions and water-to-cement ratios. At −20 °C, the normalized resistivity values were approximately 3.0–3.7, indicating resistivities of 3.0–3.7 times the corresponding values at 20 °C. At 80 °C, the normalized values decreased to approximately 0.18–0.30. Although some scatter was observed among the groups at individual temperatures, the relative changes were generally similar, with no consistent increase or decrease in the temperature response as either the CCSF volume fraction or the water-to-cement ratio increased. These results indicate that, within the investigated parameter ranges, CCSF volume fraction and water-to-cement ratio primarily affected the absolute resistivity values and had only limited effects on the relative response of resistivity to temperature.

### 3.5. Effect of Applied Voltage on the Electrical Properties of Concrete

**Figure 16:**
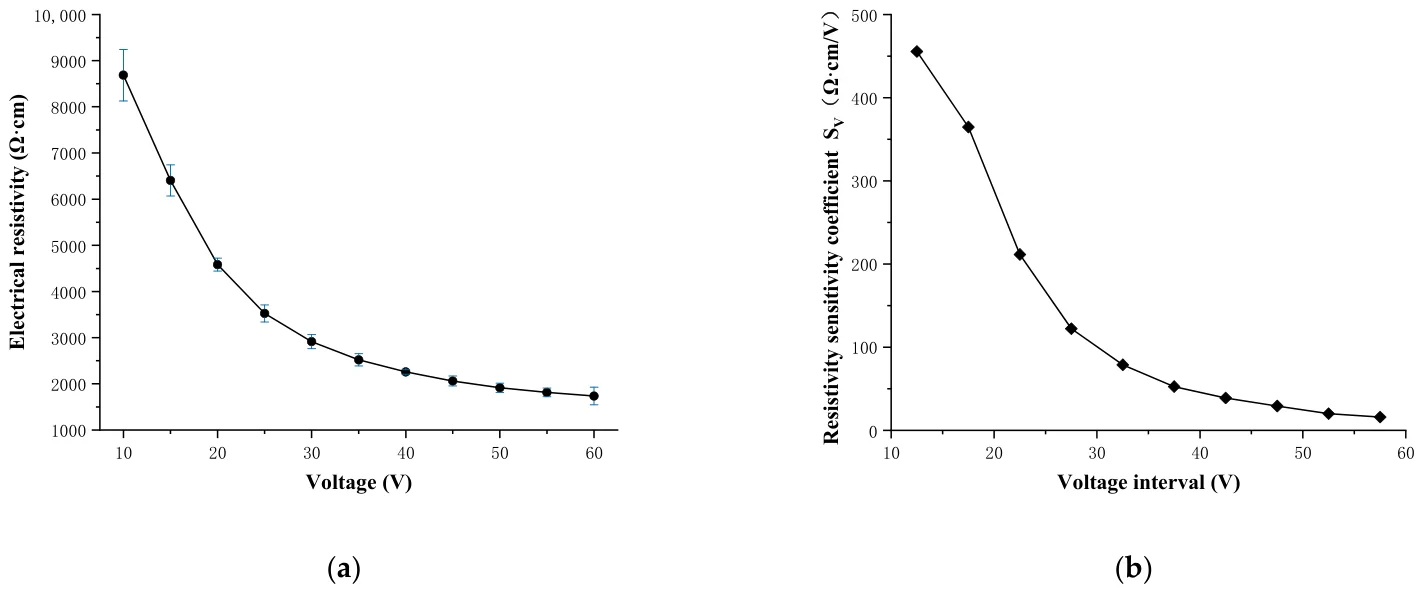
Effect of applied voltage on the electrical properties of concrete (W/C = 0.45 and CCSF volume fraction = 1.0%): (**a**) Relationship between specimen electrical resistivity and applied voltage, *p* = 8.287 × 10^−21^. (**b**) Relationship between the resistivity sensitivity coefficient S_V_ and applied voltage.

The experimental results showing the effect of applied voltage on the electrical resistivity of concrete are presented in Figure 16a. With all other test conditions held constant, increasing the applied voltage from 10 V to 60 V reduced the measured resistivity of CCSF concrete from 8684.89 Ω·cm to 1734.92 Ω·cm, corresponding to an overall reduction of 80.02%. This decrease was nonlinear, with a rapid reduction at low voltages followed by a progressively smaller decrease. When the applied voltage increased from 10 V to 25 V, the resistivity decreased to 3525.68 Ω·cm, representing a reduction of 59.40%. A further increase to 40 V reduced the resistivity to 2257.23 Ω·cm, the decrease accounts for 18.25% of the total decrease between 10 and 60 V. These results indicate that the influence of applied voltage on measured resistivity differed considerably across voltage intervals.

The sensitivity coefficient of electrical resistivity with respect to applied voltage, S_V_, is shown in Figure 16b. The coefficient decreased continuously with increasing applied voltage, with an overall reduction of 96.49% across the investigated range. Within the 10–15 V interval, S_V_ was 455.54 Ω·cm/V, indicating that the measured resistivity was particularly sensitive to changes in applied voltage at low voltages. Within the 25–30 V interval, S_V_ decreased to 122.23 Ω·cm/V, representing a reduction of 73.2%, and eventually reached 16.01 Ω·cm/V within the 55–60 V interval.

**Figure 17:**
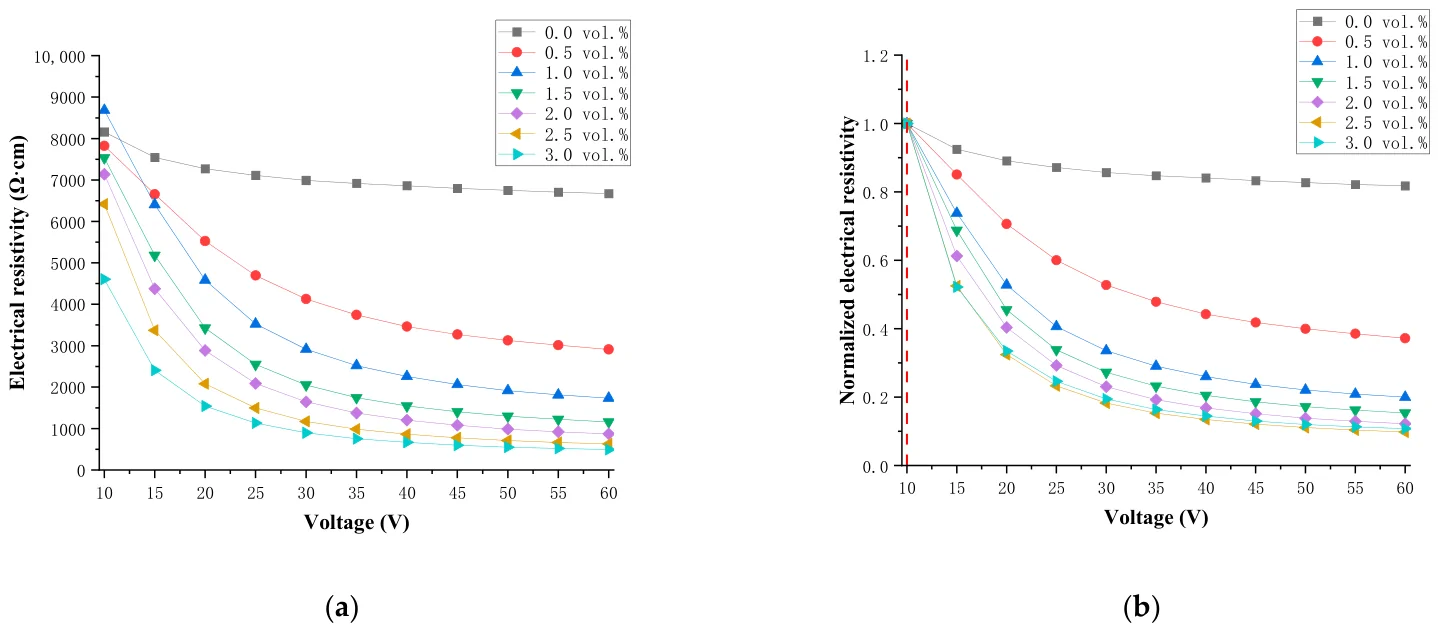
Relationship between electrical resistivity and applied voltage at different CCSF volume fractions (W/C = 0.45): (**a**) Relationship between specimen electrical resistivity and applied voltage at different CCSF volume fractions. (**b**) Relationship between normalized electrical resistivity and applied voltage at different CCSF volume fractions.

The resistivity responses of specimens with different CCSF volume fractions are shown in Figure 17a. The response to applied voltage differed considerably among the specimens. Specimens without CCSF exhibited only a small change in resistivity, with a reduction of 18.30% over the investigated voltage range. After CCSFs were incorporated, the resistivity decreased much more rapidly with increasing applied voltage.

Because the absolute resistivity values differed substantially among specimens with different CCSF volume fractions, the original curves did not allow direct comparison of their relative changes. The data were therefore normalized by the reference resistivity at 10 V, as shown in Figure 17b. The results indicate that CCSF volume fraction strongly influenced the relative response of resistivity to applied voltage. At 60 V, the normalized resistivity of specimens without CCSF remained approximately 0.82, whereas the values for specimens with CCSF volume fractions of 1%, 2%, and 3% decreased to approximately 0.20, 0.12, and 0.11, respectively. The relative reduction in resistivity induced by increasing applied voltage became more pronounced as the CCSF volume fraction increased, although the differences between groups at higher fiber volume fractions became smaller. These results indicate that incorporating CCSF substantially increased the sensitivity of resistivity to applied voltage, while the additional increase in sensitivity diminished at higher CCSF volume fractions.

**Figure 18:**
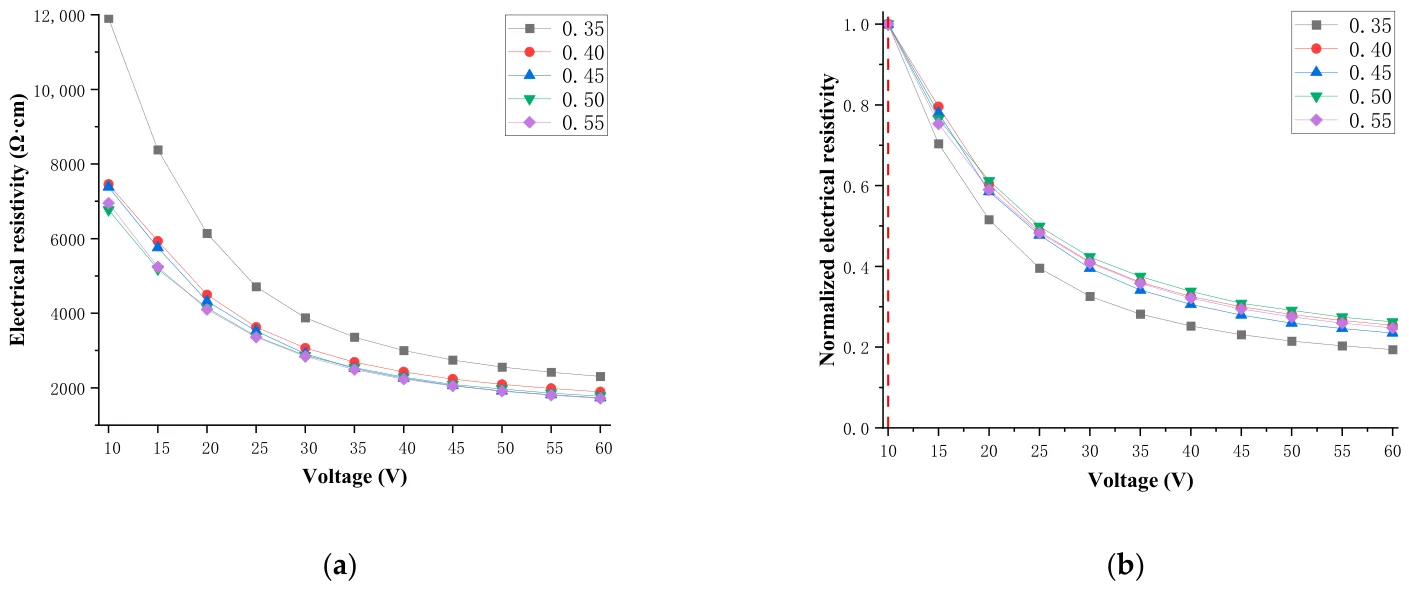
Relationship between electrical resistivity and applied voltage at different water-to-cement ratios (CCSF volume fraction = 1.0%): (**a**) Relationship between specimen electrical resistivity and applied voltage at different water-to-cement ratios. (**b**) Relationship between normalized electrical resistivity and applied voltage at different water-to-cement ratios.

In contrast, specimens with different water-to-cement ratios generally exhibited similar resistivity responses to applied voltage. As shown in Figure 18, the specimens with a water-to-cement ratio of 0.35 exhibited a comparatively stronger response. Their poorer casting quality resulted in less continuous conductive pathways and less stable contacts, making their electrical behavior more sensitive to changes in the applied electric field. Once the water-to-cement ratio reached 0.40 or higher, the mixture provided adequate conditions for specimen casting and compaction. Consequently, the normalized curves nearly overlapped, indicating broadly consistent resistivity responses to applied voltage among these groups.

CCSFs themselves exhibit ohmic behavior, and their resistance is therefore not substantially affected by applied voltage. Meanwhile, the overall resistivity reduction in specimens without CCSF was only 18.30%, and the normalized resistivity responses were broadly similar across specimens with different water-to-cement ratios. These observations indicate that the concrete matrix had a limited response to changes in applied voltage. The voltage response of CCSF concrete is therefore primarily associated with interfacial charge transport and changes in conductive pathways introduced by the fibers, rather than with changes in ionic conduction within the matrix or electronic conduction within the fibers themselves.

As the applied voltage increases, the stronger electric field promotes charge transport across the fiber–matrix interfaces, resulting in a nonlinear electrical response characterized by a continuous decrease in measured resistivity [29,30]. At higher voltages, the dominant conductive pathways gradually stabilize, and the additional enhancement of interfacial charge transport becomes smaller. Consequently, the decrease in resistivity becomes progressively less pronounced. The responses observed at different CCSF volume fractions further support this interpretation. Increasing the CCSF volume fraction increases the total fiber–matrix interfacial area and reduces the spacing between fibers, thereby facilitating interfacial charge transport and improving the connectivity of conductive pathways. The voltage response therefore becomes stronger. With further increases in CCSF volume fraction, however, the conductive network becomes more fully developed, and the additional enhancement of the voltage response gradually diminishes.

## 4. Conclusions

This study investigated the effects of CCSF volume fraction, water-to-cement ratio, temperature, and applied voltage on the electrical properties of concrete. Sensitivity coefficients and normalization were used to characterize the electrical responses to these factors, providing a basis for optimizing the resistivity measurement method. The main conclusions are as follows:

(1) CCSF volume fraction was a major factor governing the electrical properties of concrete, whereas the influence of the water-to-cement ratio differed between distinct regimes. Increasing the CCSF volume fraction from 0% to 3.0% reduced resistivity by 87.18%, with a transition between the two response regimes at approximately 0.8%. The influence of the water-to-cement ratio was most pronounced within the 0.35–0.40 interval. Once adequate conditions for specimen casting and compaction were achieved, further increases in the water-to-cement ratio had a substantially weaker effect on resistivity.

(2) Temperature substantially affected electrical resistivity. Increasing the temperature from −20 °C to 80 °C reduced resistivity by 92.99%. Specimens with different CCSF volume fractions and water-to-cement ratios exhibited broadly similar temperature dependence after normalization. These results indicate that CCSF volume fraction and water-to-cement ratio primarily affected the absolute resistivity values and had limited effects on the relative response to temperature.

(3) Applied voltage substantially affected measured resistivity, and the relative response depended on CCSF volume fraction. Increasing the applied voltage from 10 V to 60 V reduced resistivity by 80.02%. Increasing the CCSF volume fraction markedly enhanced the voltage response of normalized resistivity, whereas the water-to-cement ratio had little influence once adequate casting and compaction were achieved. This response was primarily associated with charge transport across the fiber–matrix interfaces and changes in conductive pathways.

(4) The optimized resistivity measurement method effectively reduced the influence of differences in applied voltage on the measurement results. Appropriate test parameters were selected by considering measurement stability, polarization effects, and Joule heating. The DC two-electrode method enabled direct control of the voltage across the measurement section, thereby avoiding additional differences in measured resistivity arising from inconsistent actual voltages across specimens and improving the comparability of electrical resistivity measurements.

## Figures and Tables

**Table 1:** Characteristic parameters of CCSF.

Length (mm)	Diameter (mm)	Aspect Ratio	Density (g/cm^3^)	Tensile Strength (MPa)	Elastic Modulus (GPa)	Elongation (%)	Electrical Resistivity (Ω·m)
13	0.22	59	7.8	2850	200	2	1.5 × 10^−7^

**Table 2:** Characteristic parameters of the 316 stainless-steel electrode.

Square Opening Size (mm)	Wire Diameter (mm)	Effective Height (mm)	Effective Width (mm)	Elastic Modulus (GPa)	Electrical Resistivity (Ω·m)
10	1.0	100	89	193	7.5 × 10^−7^

**Table 3:** Specific mix proportion for CCSF concrete.

Group	Cement (kg/m^3^)	Water (kg/m^3^)	Coarse Aggregate (kg/m^3^)	Fine Aggregate (kg/m^3^)	CCSF (kg/m^3^)
A0.0	500	225	900	600	0
A0.1	500	225	900	600	7.8
A0.2	500	225	900	600	15.6
A0.3	500	225	900	600	23.4
A0.4	500	225	900	600	31.2
A0.5	500	225	900	600	39
A0.6	500	225	900	600	46.8
A0.8	500	225	900	600	62.4
A1.0	500	225	900	600	78
A1.5	500	225	900	600	117
A2.0	500	225	900	600	156
A2.5	500	225	900	600	195
A3.0	500	225	900	600	234
B35	500	175	900	600	78
B40	500	200	900	600	78
B50	500	250	900	600	78
B55	500	275	900	600	78

## Data Availability

The data provided in the article can be obtained from the corresponding author.
